# A Mandibular Premolar With Varied Configuration: A Case Report

**DOI:** 10.7759/cureus.94646

**Published:** 2025-10-15

**Authors:** Basanthi Choudhary, Naresh Gaddala, Shekhar Kamishetty, Syed Waseem Uddin, Darsha Nirupama

**Affiliations:** 1 Conservative Dentistry and Endodontics, Sri Sai College of Dental Surgery and Hospital, Vikarabad, IND

**Keywords:** canal configuration, cone beam computed tomography (cbct), mandibular first premolar, root canal treatment, thermoplasticized gutta-percha

## Abstract

The root canal system anatomy significantly influences the outcome of endodontic treatment. Mandibular premolar teeth are known for their considerable variations in root canal morphology. Typically, the mandibular first premolar has a straight forward anatomy with one root and one canal. However, the presence of a varied configuration of mandibular premolars is documented in the literature as it is considered an "enigma to the endodontist." This article presents a case of a successful nonsurgical endodontic treatment of a mandibular premolar with a varied configuration.

## Introduction

The main objective of endodontic treatment is to achieve adequate mechanical and chemical cleaning of the entire root canal system, followed by a three-dimensional seal using an inert material and the establishment of a proper coronal seal. Common reasons for failure in endodontic therapy include improper canal instrumentation, missed canals, and incomplete filling of the root canal. These factors can lead to unsuccessful treatment outcomes and may trigger acute flare-ups either during or after the procedure. Identifying all the canals is essential to prevent such failures.

A comprehensive understanding of root canal anatomy is therefore essential for successful outcomes. As emphasized by Slowey, mandibular premolars are particularly difficult to manage in endodontic treatment due to their intricate anatomical variations [1]. These complexities often result in increased instances of treatment failure and flare-ups. The unique morphology of these teeth requires practitioners to be especially vigilant, as variability in root canal anatomy presents significant challenges during endodontic procedures.

Anatomical studies have shown that the presence of three-rooted mandibular first premolars is uncommon, with an estimated occurrence of about 0.2% [2]. This highlights the need for careful examination when treating these teeth to ensure that all potential canals are identified and managed appropriately.

This article presents a case of a mandibular premolar with a varied root canal configuration, which was classified according to the Ahmed et al., and was successfully managed with nonsurgical endodontic treatment [3]. The treatment underscores the importance of accurate identification in addressing the unique anatomical variations present in these teeth, which can significantly impact the effectiveness of endodontic procedures. By employing thorough cleaning and sealing techniques, this case demonstrates how understanding root canal anatomy can lead to favorable outcomes in challenging endodontic scenarios.

## Case presentation

**Figure 1:**
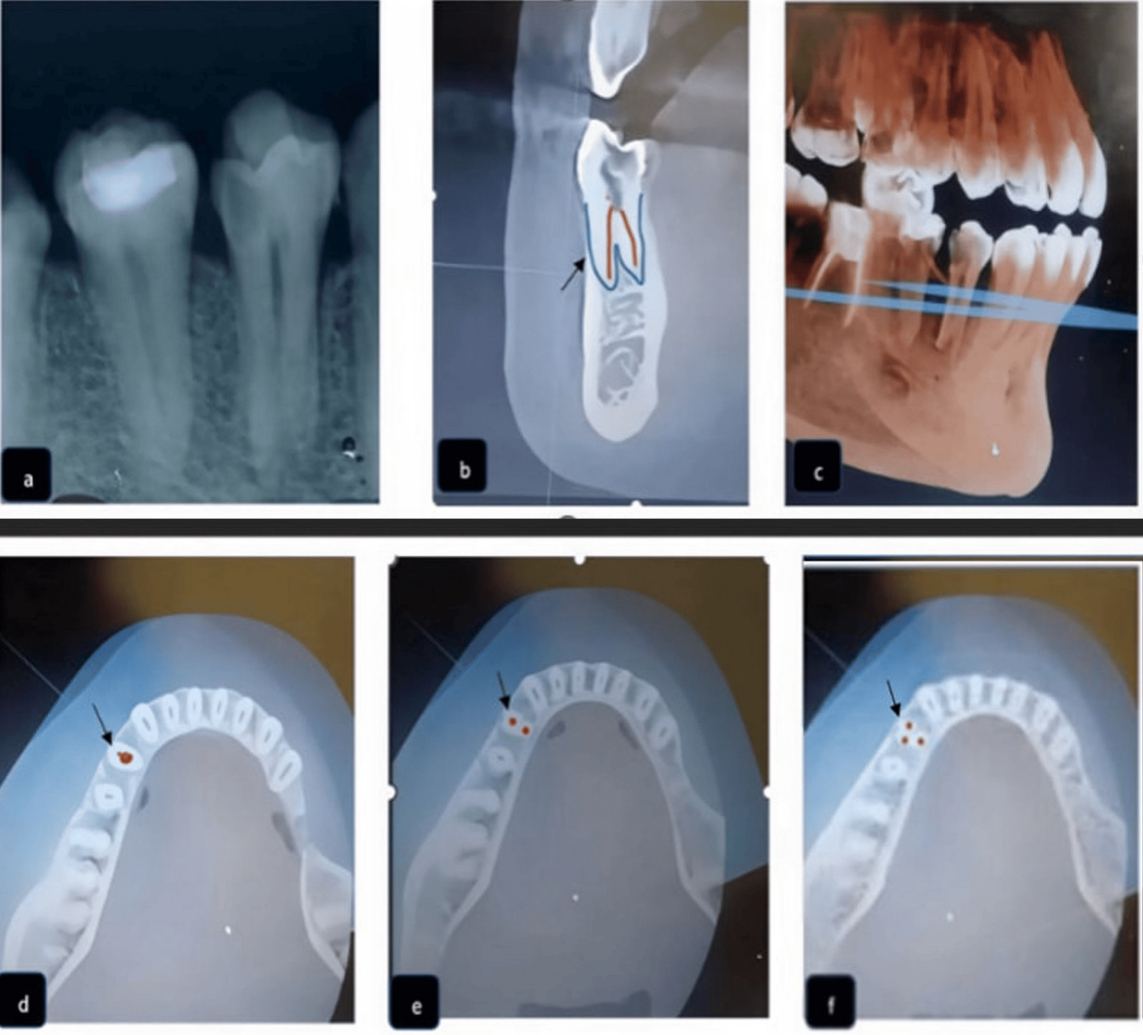
Pre-operative radiographic and CBCT images. (a) Pre-operative radiograph; (b, c) pre-operative CBCT sagittal cross-sections, here the blue line represents the root border and the red line represents the canal configuration; (d) axial view at the coronal third; (e) axial view at the middle third; (f) axial view at the apical third. In (d-f), red dots represent the canal configuration, and arrows pointing to the dots indicate the labial side. CBCT: cone beam computed tomography

This patient was treated in the Department of Conservative Dentistry and Endodontics, Sri Sai College of Dental Surgery, Vikarabad. A 28-year-old female patient was referred to the Department of Conservative Dentistry and Endodontics with the chief complaint of pain in the lower right back tooth region for the past one month. Clinical examination revealed proximal caries in the mandibular right first premolar (tooth #44), along with mild tenderness on percussion. The patient's medical history was unremarkable. The pre-operative diagnostic radiograph revealed a disto-occlusal radiolucency approaching the pulp, with an abnormal canal configuration (Figure 1, panel a). Vitality testing was performed using a cold test (ROEKO Endo-Frost; Cuyahoga Falls, OH: Coltene Inc.), which showed a delayed response.

Based on the pre-operative radiograph, cone beam computed tomography (CBCT) was advised for the identification of canals. Images were taken using SCANORA 3Dx (Tuusula, Finland: SOREDEX) with standard operating specifications (90 kV and 10 mA), and were used for the analysis of additional canals and root canal morphology in both jaws, using a medium field of view (field of view {FOV}=80×100), with a standard resolution mode (voxel size of 0.25 mm) (Figure 1, panels b-f). The allocated complete scan time comprised a 360° rotation of the X-ray receptor assembly around the static patient, which revealed the presence of two roots with a three-canal configuration. This anatomical variation could not be classified according to Vertucci's classification system; therefore, it was categorized according to the new classification proposed by Ahmed et al. as 2441M1D [3].

**Figure 2:**
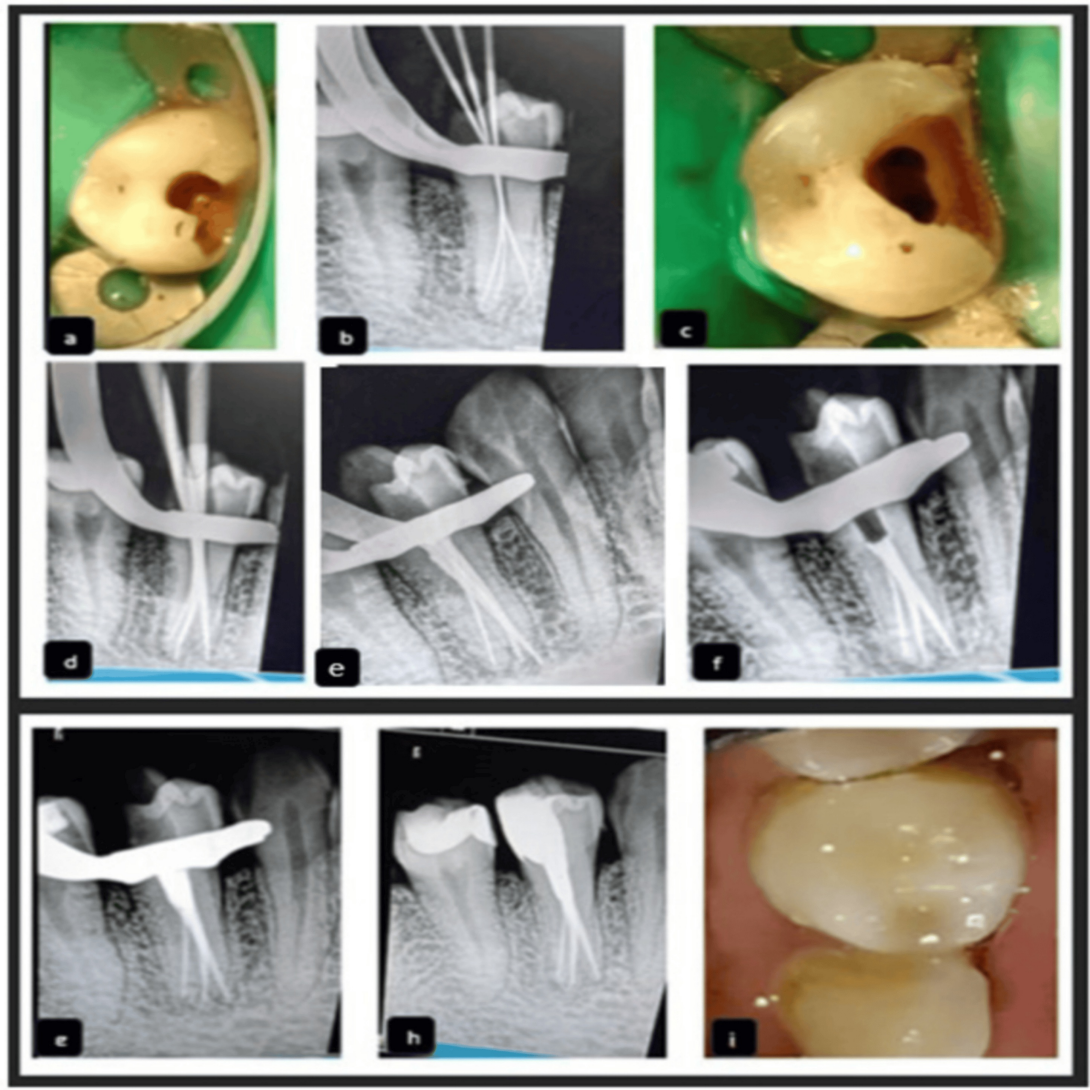
Clinical and radiographic sequence. (a) Pre-operative clinical image, (b) working length radiograph, (c) clinical view after caries excavation, (d) master cone radiograph, (e) obturation completed, (f) clinical view after crown shearing up to trifurcation level, (g) backfilling of the remaining canal, (h) composite post-endodontic restoration, and (i) post-operative clinical image.

A diagnosis of symptomatic irreversible pulpitis with symptomatic apical periodontitis was made for tooth #44 (Figure 2, panel a). Root canal treatment was planned and performed with magnification (dental loupes). Local anesthesia was administered using 2% lignocaine containing 1:200,000 epinephrine (Xylocaine; Bengaluru, India: AstraZeneca Pharma India Ltd.). Access opening was performed using an endo-access bur, and the canal orifices were enlarged up to Gates Glidden drill size 3 (Utsunomiya, Japan: Mani, Inc.) (Figure 2, panel c).

The working length was determined using a #10 K-file with an electronic apex locator (Root ZX; Tokyo, Japan: J. Morita Corp.) and confirmed radiographically (Figure 2, panel b). Biomechanical preparation was carried out using ProTaper Gold rotary files (Ballaigues, Switzerland: Dentsply Sirona) up to size F2 at a speed of 300 RPM and a torque of 1.5 N·cm. Irrigation was performed using normal saline, 5.25% sodium hypochlorite, and 17% ethylenediaminetetraacetic acid (EDTA). Calcium hydroxide intra-canal medicament (RC Cal; Thane, India: Prime Dental Products Pvt. Ltd.) was placed, and the access cavity was sealed with Cavit (DenTemp; New Delhi, India: Waldent). At the follow-up appointment one week later, the patient was asymptomatic. The calcium hydroxide medicament was removed using copious irrigation with saline and 5.25% sodium hypochlorite. The final irrigant used was 2% chlorhexidine solution.

The canals were dried with sterile paper points, and obturation was completed using the single cone technique (Figures 2, panels d-f). As the coronal third of the canal was wide, to achieve a three-dimensional hermetic seal, the gutta-percha was sheared off at the level of trifurcation. Backfilling of the remaining canal space was performed using thermoplasticized gutta-percha (Figures 2, panels g and h) with the Calamus Dual System (Ballaigues, Switzerland: Dentsply Maillefer), followed by a composite post-endodontic restoration (Figure 2, panel i, Table 1).

**Table 1:** Technical information and treatment sequence for the case reported in this study. EDTA: ethylenediaminetetraacetic acid

Sequence	First appointment	Second appointment
1	Local anesthesia 2% lignocaine1:200,000 epinephrine	Cavit was removed
2	Rubber dam isolation and access opening	Calcium hydroxide medicament was removed
3	Canal orifices were enlarged with Gg drills, size 3	Irrigation done with 5 mL of saline
4	Working length #10 K-file (electronic apex locator) and confirmed with periapical radiography	Irrigation done with 5 mL of 5.25% NaOCl
5	Biomechanical preparation was carried out using ProTaper Gold rotary files up to file F3	Irrigation was done with 5 mL of saline
6	Irrigation was performed using 5 mL of saline	Irrigation done with 2% chlorhexidine solution
7	Irrigation with 5 mL of 5.25% NaOCl	Single cone obturation done till the trifurcation
8	Irrigation with 5 mL of saline	Backfilling of the remaining canal space was performed using thermoplasticized gutta-percha technique
9	Irrigation with 5 mL of EDTA	Composite post-endodontic restoration done
10	Irrigation with 5 mL of saline	-
11	Calcium hydroxide intra-canal medicament (RC Cal) was placed	-
12	Access cavity was sealed with Cavit (DenTemp; New Delhi, India: Waldent)	-

## Discussion

Mandibular premolars normally have one root and one canal, as reported by many authors, including Pineda and Kuttler [2-5]. However, anatomical variations of mandibular premolars are documented in the literature. More commonly encountered variations include teeth with two roots and/or canals, as mentioned by Green and Shapira and Delivanis [6,7]. Rare aberrations, such as three [8], four [9], or even five root canals, have also been documented [10]. Vertucci and Zillich and Dowson reported the occurrence of three canals in mandibular first premolars in 0.5% and 0.4% of cases, and in second premolars in 0.0% and 0.4% of cases, respectively [5,11].

Mandibular premolars, particularly those with complex anatomy, present a significant endodontic challenge. The presence of multiple canals further complicates the procedure. Understanding and managing these variations plays a significant role in the success of endodontic treatment. In the present case, a single wide orifice with three canals terminating in three separate apical foramina was observed.

Bifurcations or trifurcations deeper within the root further complicate visibility and access. Several clinical signs can help identify the presence of additional canals. For instance, suspicion should arise when the pulp chamber does not align in its typical buccolingual relationship. If the pulp chamber deviates from its normal configuration, appearing triangular or wider in the mesiodistal plane, the possibility of multiple canals should be considered.

Cone beam computed tomography (CBCT) offers significant advantages for detecting abnormalities or hidden canals. It provides high-resolution 3D imaging of tooth anatomy that is often missed with a 2D X-ray. The use of magnification also aids in locating additional canals. Tactile exploration of all the walls of the main canal(s) with a pre-curved #08 or #10 International Organization for Standardization (ISO) K-file is recommended to detect any “catch,” which may indicate the presence of an additional canal.

In the present report, the use of tactile sensation, magnification with dental loupes, and radiographic imaging (CBCT) helped in locating and negotiating the extra canals. As this canal configuration does not fall under Vertucci's classification, the new classification system proposed by Ahmed et al. was considered [3].

Once all canals were prepared, obturation was performed using the single-cone technique. Gutta-percha was sheared off at the level of the trifurcation, and the remaining portion of the canal was obturated using the thermoplasticized gutta-percha technique to achieve a three-dimensional hermetic seal [12,13]. Heated gutta-percha becomes flowable, allowing it to adapt closely to the canal walls. This technique reduces voids and gaps between gutta-percha and dentinal walls, thereby providing a better apical and coronal seal and lowering the chances of reinfection. In addition, it minimizes dentinal stress and the potential risk of root fracture that may occur with excessive lateral compaction in mandibular premolars. Overall, it produces a dense, continuous obturation mass, improving the long-term stability of the root canal filling. For a proper coronal seal, post-endodontic restoration was done with composite to prevent microleakage.

## Conclusions

This report highlights the successful endodontic treatment of a mandibular first premolar with varied canal anatomy. Understanding morphological variation in pulp canal anatomy is crucial before treatment. Knowledge of the extra canals in the mandibular premolars enhances the development of comprehensive endodontic care. Utilizing magnification tools, such as dental loupes and advanced imaging techniques, such as CBCT imaging, helps identify additional canals. Proper identification and negotiation of canals ensure predictable treatment outcomes for complex mandibular premolars.
